# Supplementary figures and images for: Distinct and diverse chromatin proteomes of ageing mouse organs reveal protein signatures that correlate with physiological functions
Source: eLife. 2022 Mar 8;11:e73524. doi: 10.7554/eLife.73524 (PMC8933006; doi:10.7554/eLife.73524)

Figure 1-source data 1  
western blotting analysis

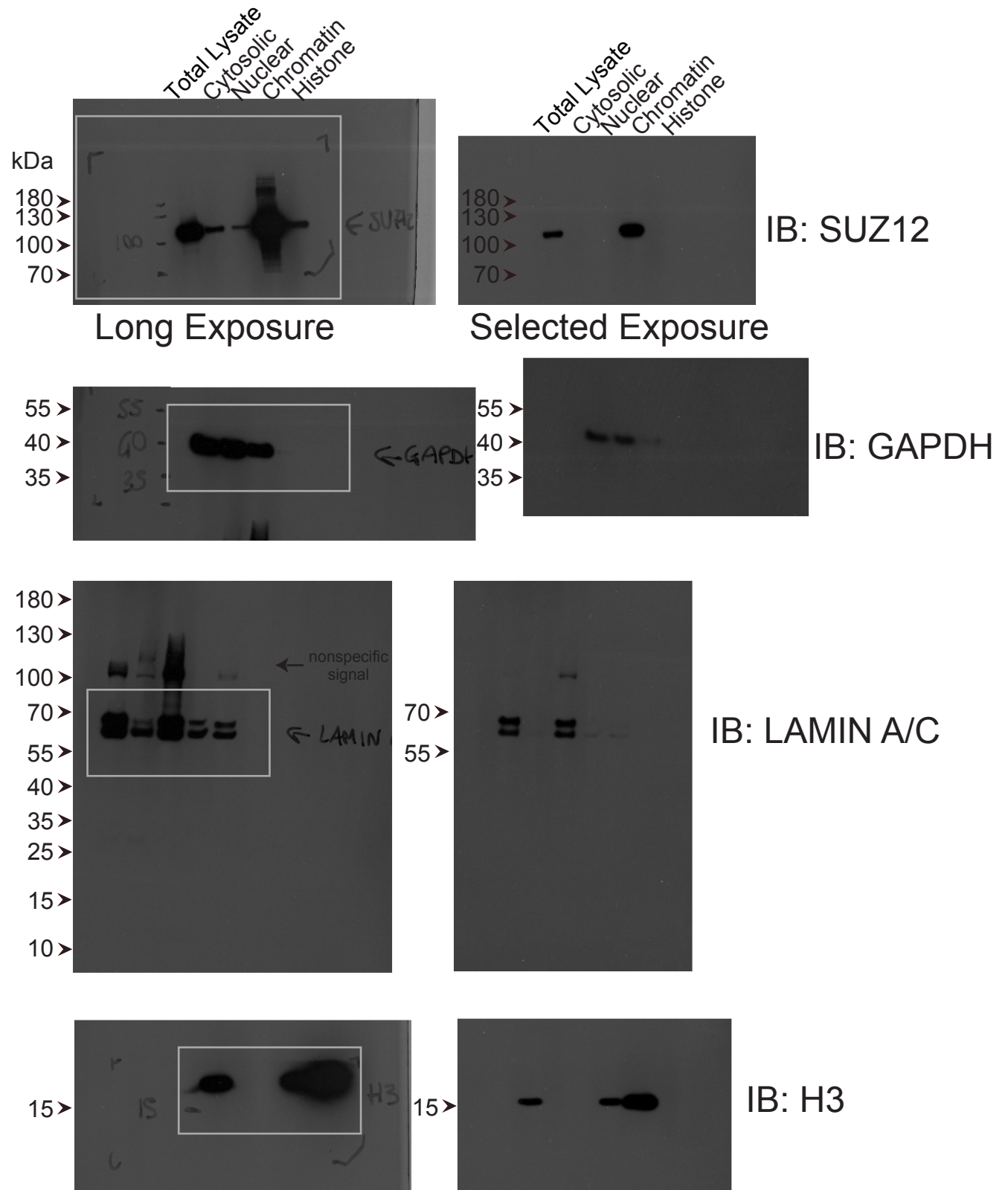

NuPAGE Gel: 4 to 12%

Supplement: Figure 1—source data 1. [file elife-73524-fig1-data1.pdf]

Figure 2-figure supplement 1-source data 1  
western blotting analysis

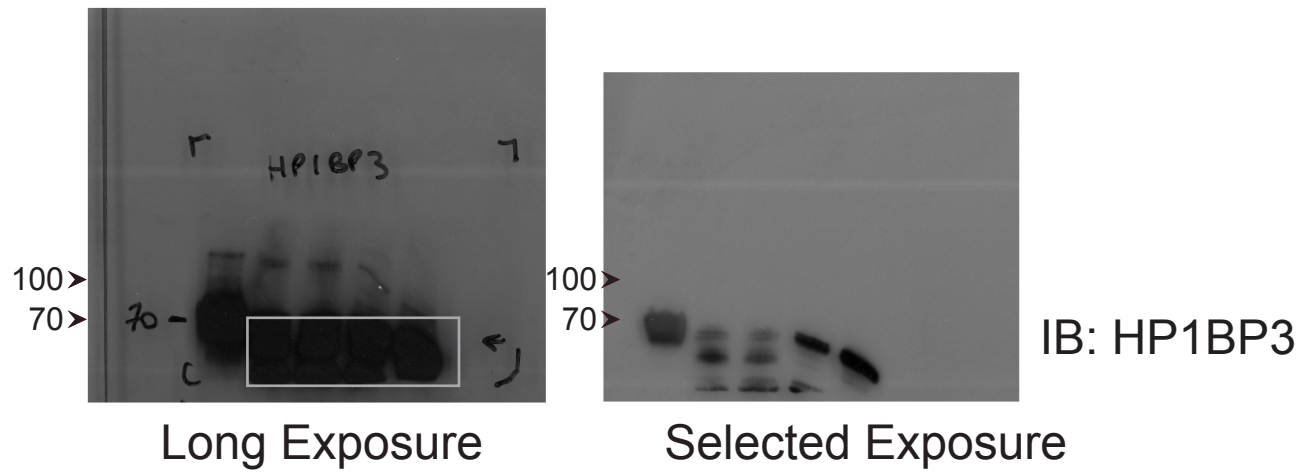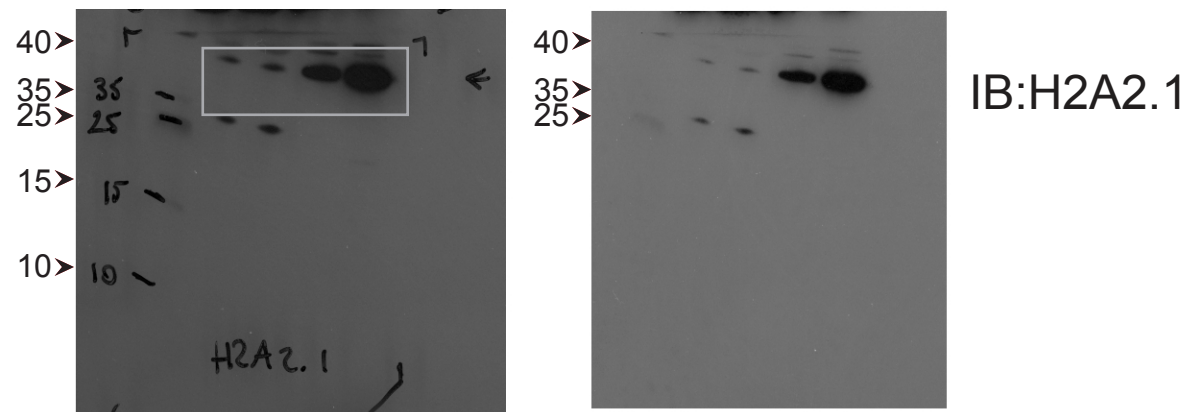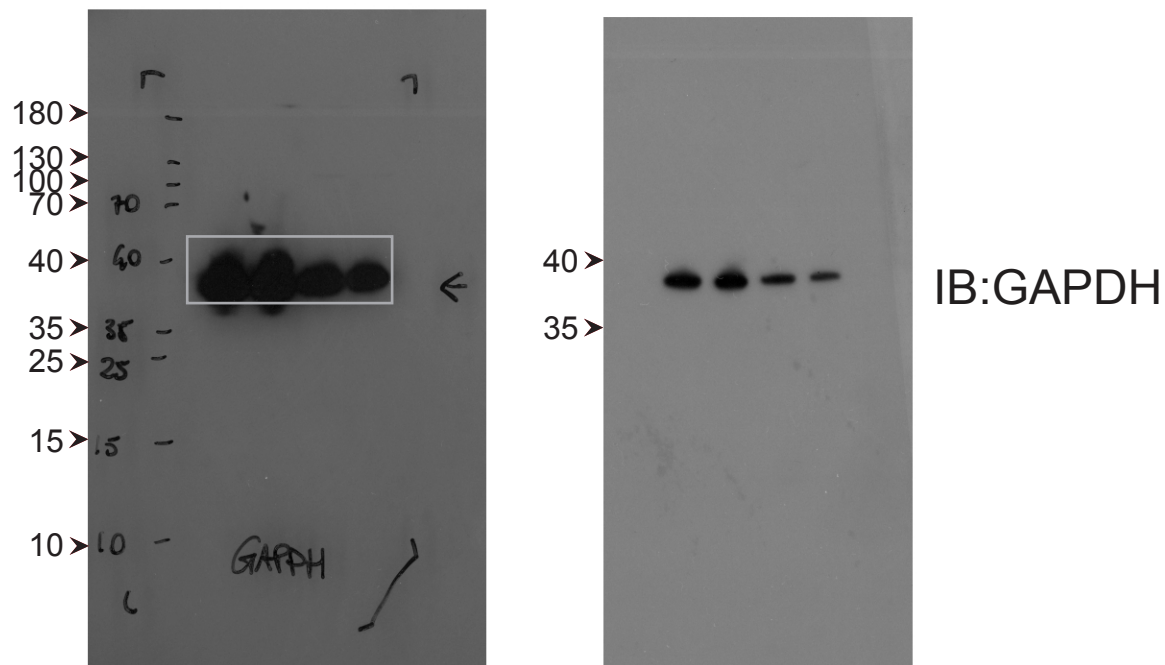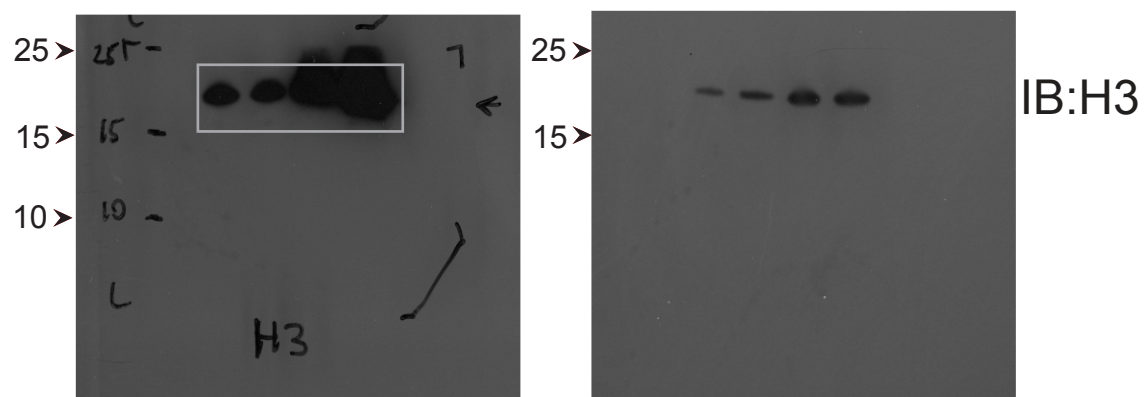

NuPAGE Gel: 4 to 12%

Supplement: Figure 1—figure supplement 2—source data 1. [file elife-73524-fig1-figsupp2-data1.pdf]

Figure 7 - source data 1- BRAIN

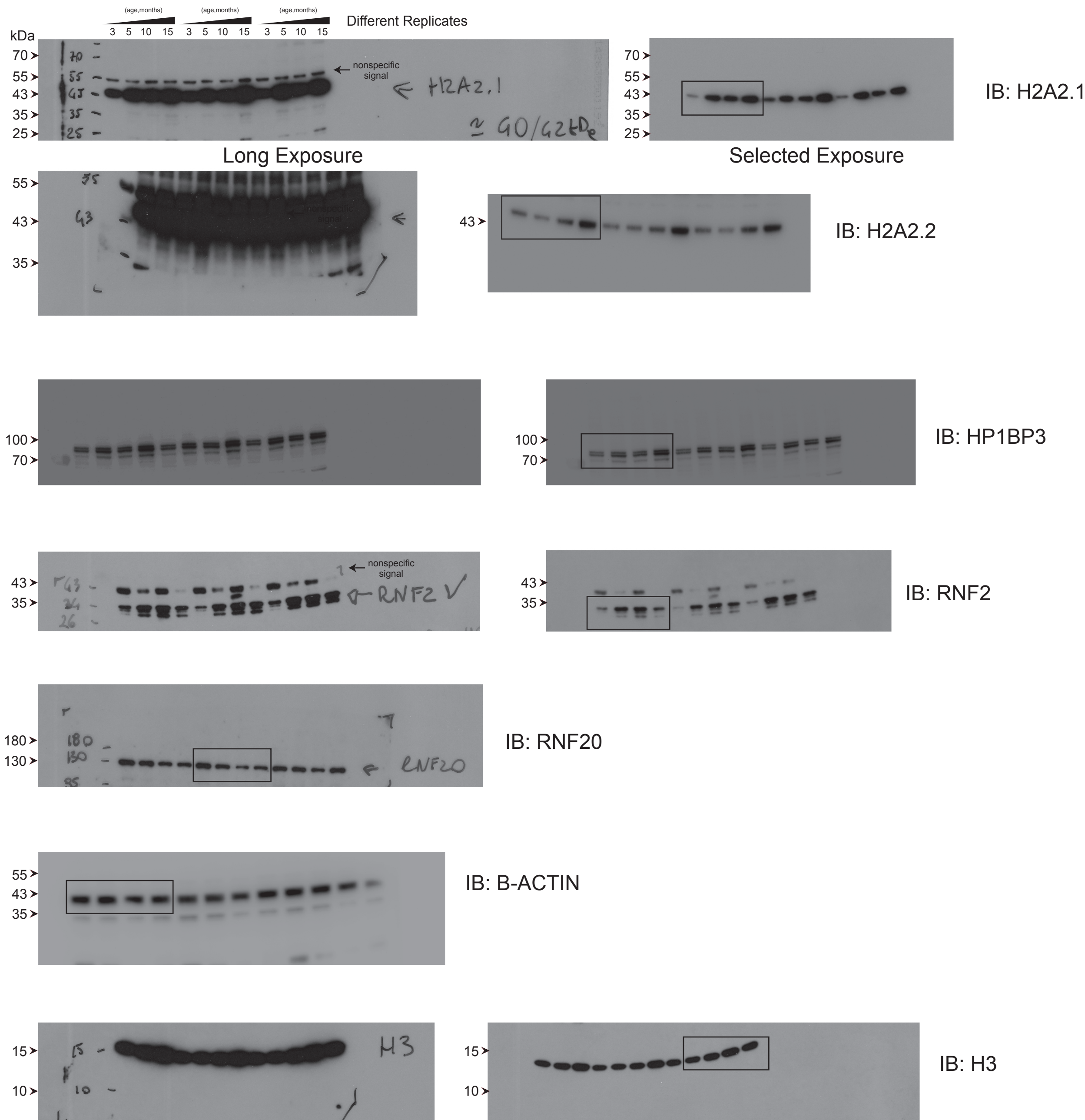

NuPAGE Gel: 4 to 12%

Supplement: Figure 7—source data 1. [file elife-73524-fig7-data1.pdf]

Figure 7- source data 2- HEART

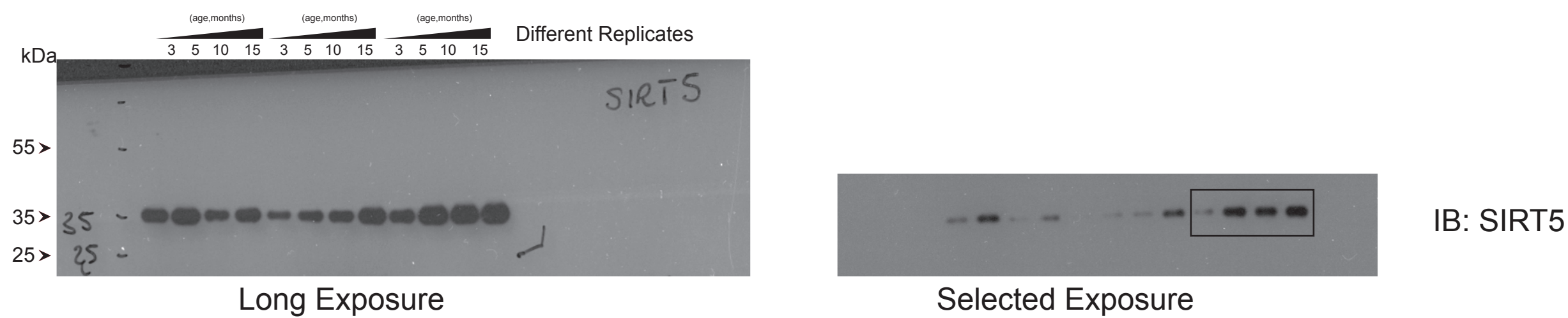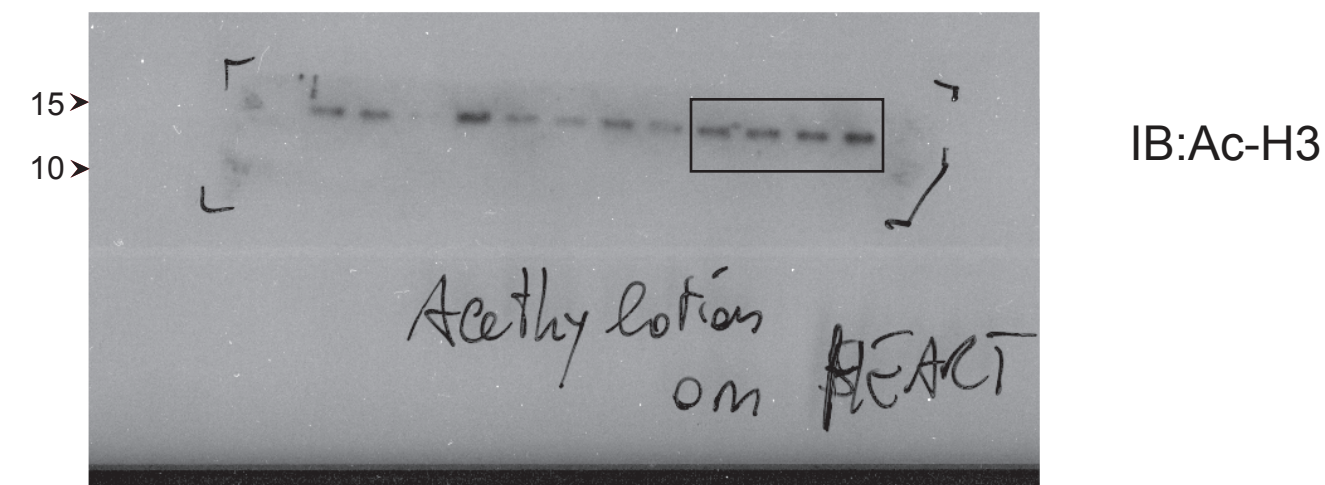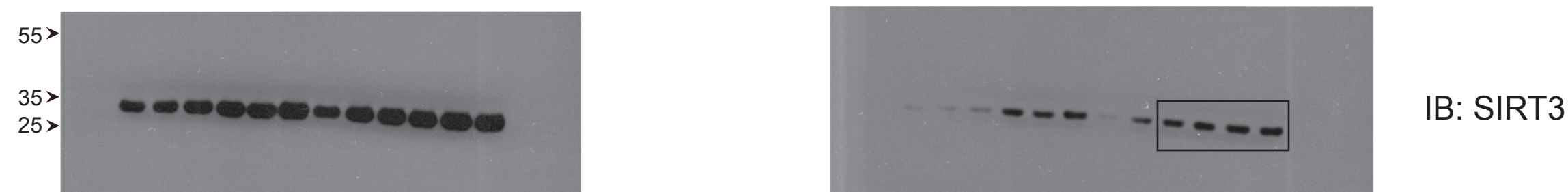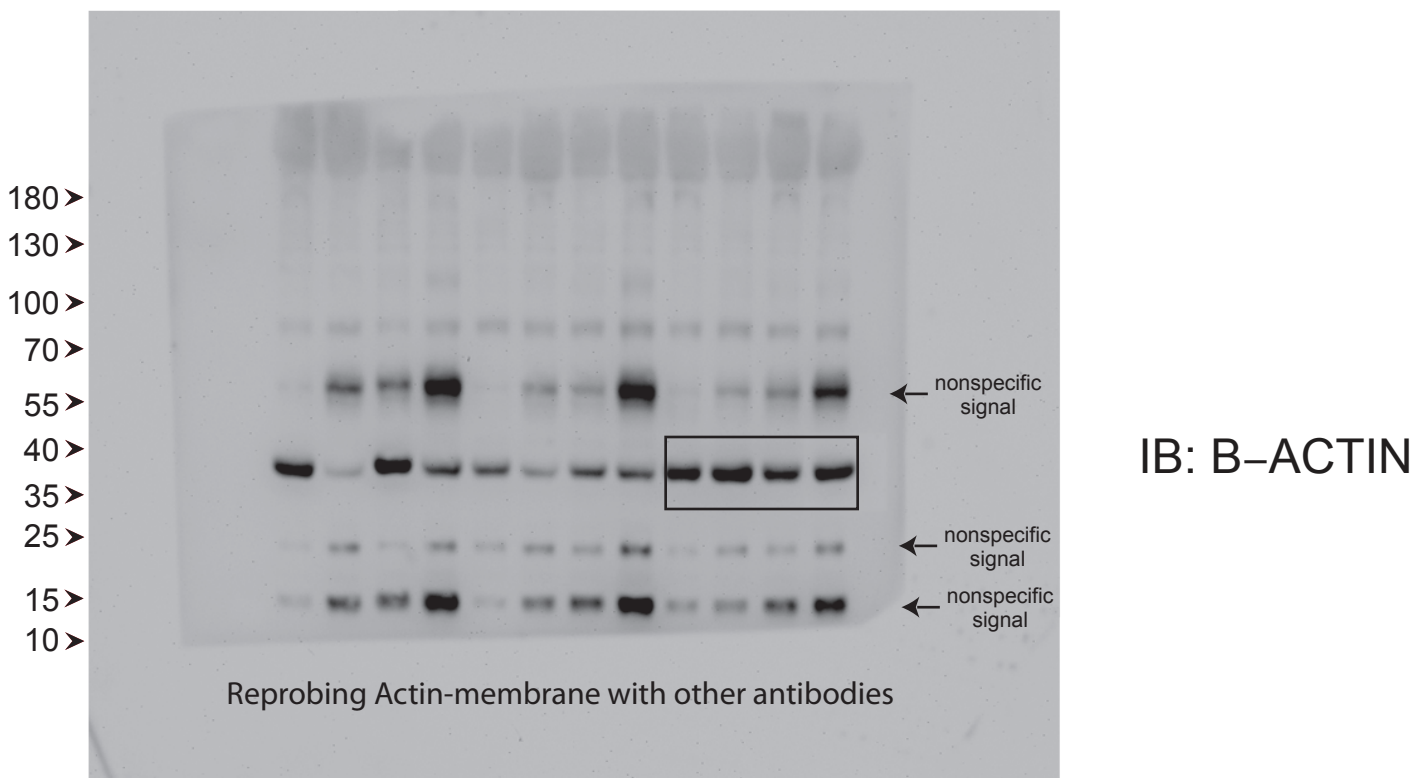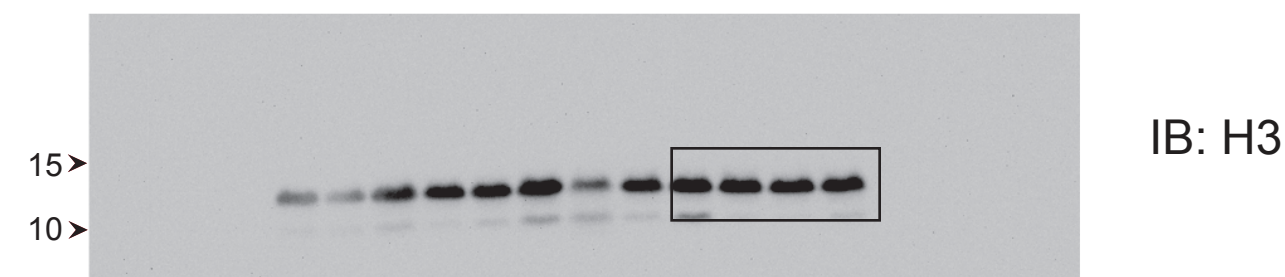

NuPAGE Gel: 4 to 12%

Supplement: Figure 7—source data 2. [file elife-73524-fig7-data2.pdf]

Figure 7 - source data 3 -KIDNEY  
western blotting analysis

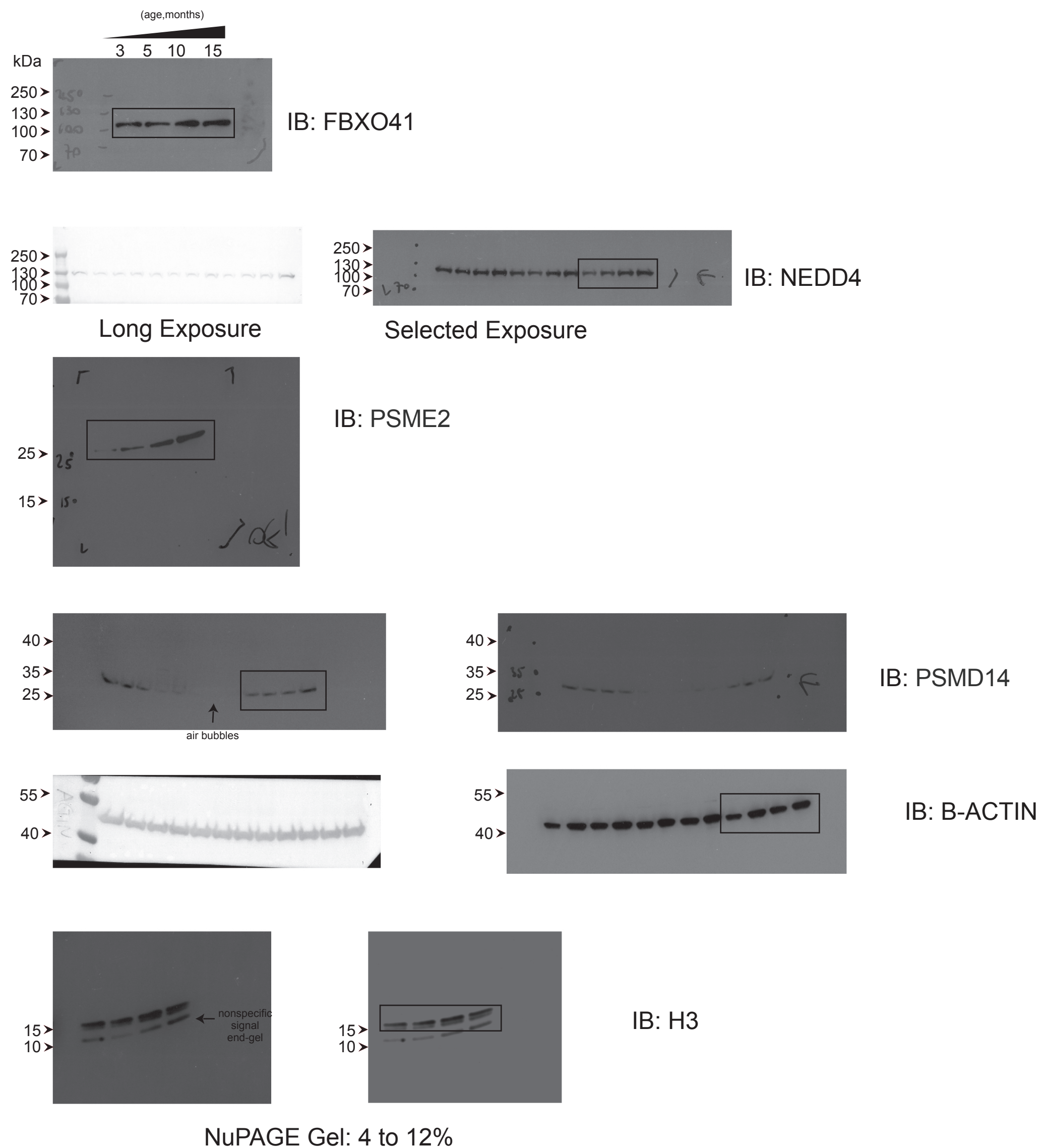

Supplement: Figure 7—source data 3. [file elife-73524-fig7-data3.pdf]

Figure 7- source data 4 - LIVER

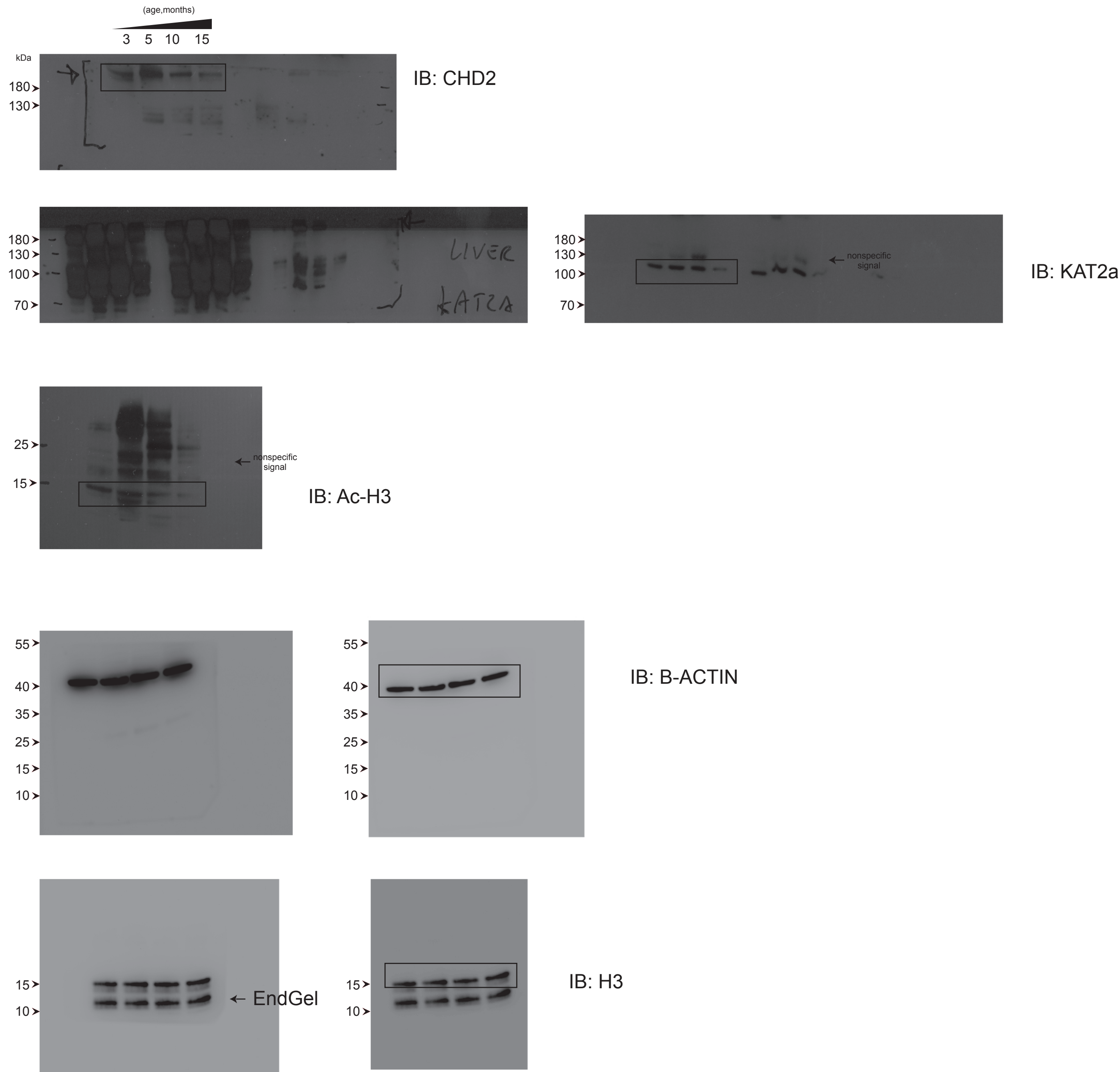

Supplement: Figure 7—source data 4. [file elife-73524-fig7-data4.pdf]

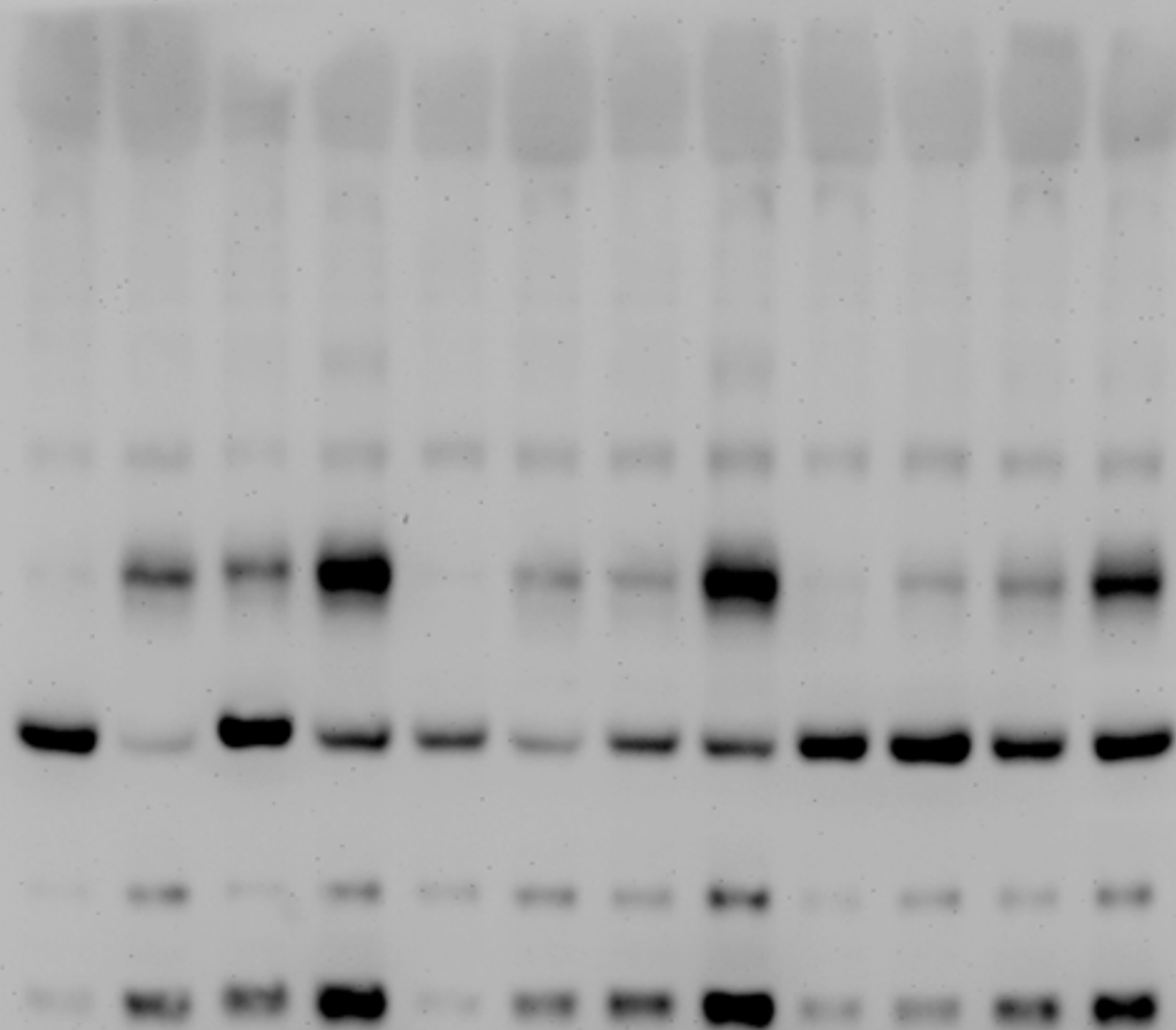

Supplement: Source data 1. [file elife-73524-data1.zip › crop_w.b/heart_7.pdf]

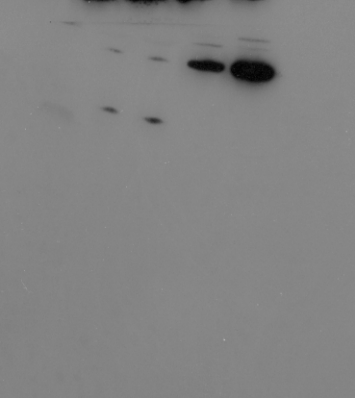

Supplement: Source data 1. [file elife-73524-data1.zip › crop_w.b/brain_4.pdf]

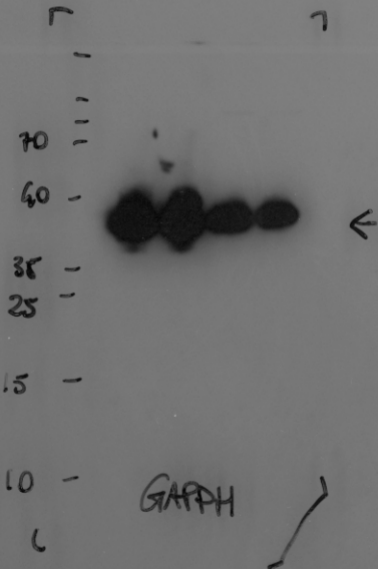

Supplement: Source data 1. [file elife-73524-data1.zip › crop_w.b/brain_5.pdf]

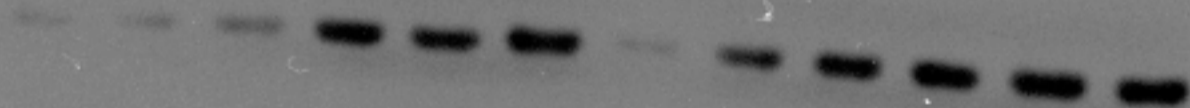

Supplement: Source data 1. [file elife-73524-data1.zip › crop_w.b/heart_6.pdf]

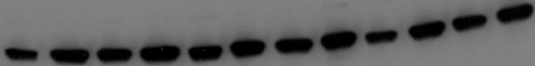

Supplement: Source data 1. [file elife-73524-data1.zip › crop_w.b/kindney_9.pdf]

M3

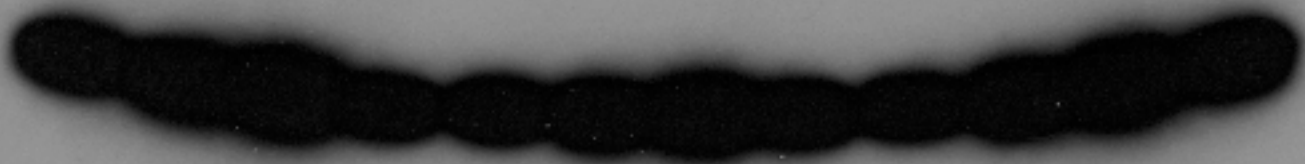

15

10

Supplement: Source data 1. [file elife-73524-data1.zip › crop_w.b/brain_18.pdf]

255-

15 -

10 -

L

H3

7

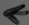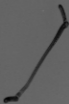

Supplement: Source data 1. [file elife-73524-data1.zip › crop_w.b/brain_7.pdf]

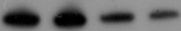

Supplement: Source data 1. [file elife-73524-data1.zip › crop_w.b/brain_6.pdf]

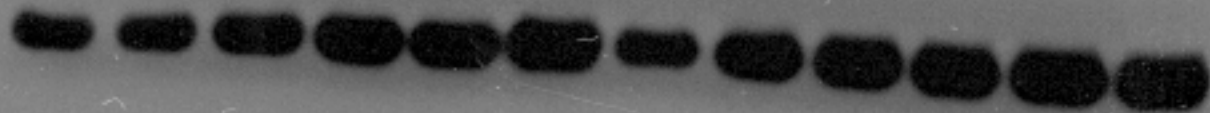

Supplement: Source data 1. [file elife-73524-data1.zip › crop_w.b/heart_5.pdf]

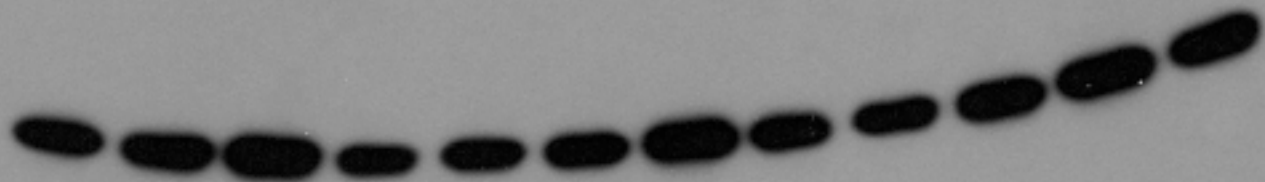

Supplement: Source data 1. [file elife-73524-data1.zip › crop_w.b/brain_19.pdf]

SIRT5

35

25

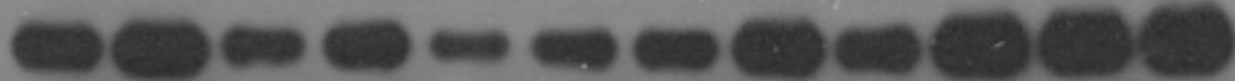

Supplement: Source data 1. [file elife-73524-data1.zip › crop_w.b/heart_1.pdf]

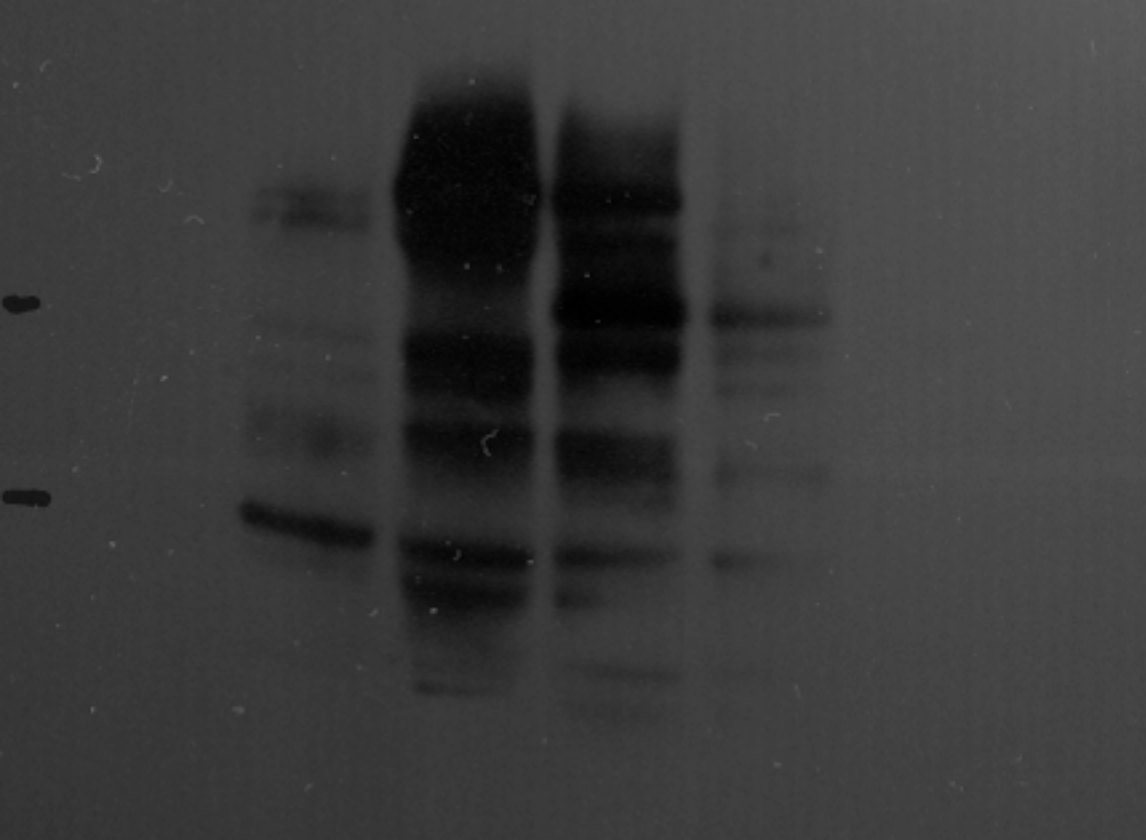

Supplement: Source data 1. [file elife-73524-data1.zip › crop_w.b/liver_4.pdf]

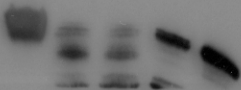

Supplement: Source data 1. [file elife-73524-data1.zip › crop_w.b/brain_2.pdf]

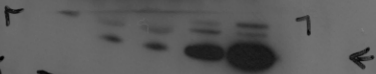

35  
25

15

10

H2A2.1

Supplement: Source data 1. [file elife-73524-data1.zip › crop_w.b/brain_3.pdf]

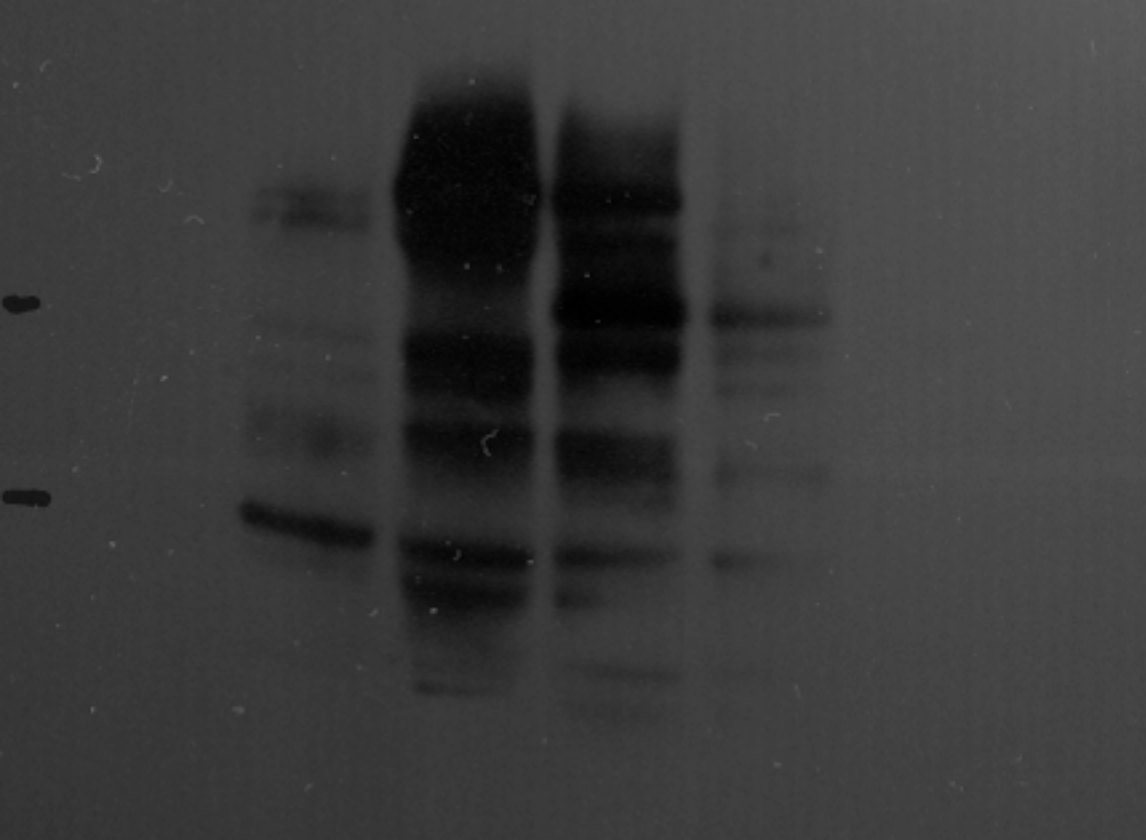

Supplement: Source data 1. [file elife-73524-data1.zip › crop_w.b/liver_5.pdf]

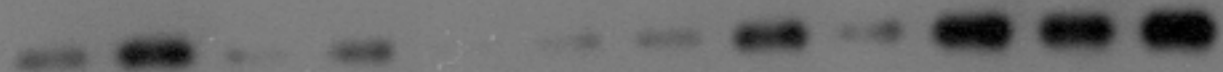

Supplement: Source data 1. [file elife-73524-data1.zip › crop_w.b/heart_2.pdf]

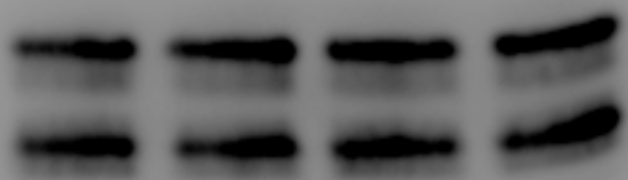

Supplement: Source data 1. [file elife-73524-data1.zip › crop_w.b/liver_7.pdf]

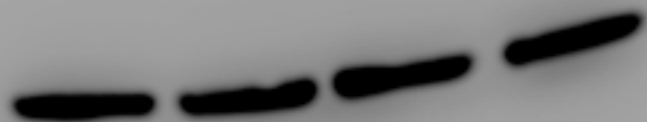

Supplement: Source data 1. [file elife-73524-data1.zip › crop_w.b/liver_6.pdf]

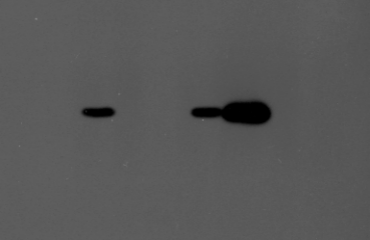

Supplement: Source data 1. [file elife-73524-data1.zip › crop_w.b/mESC_7.pdf]

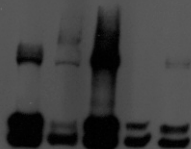

← LAMIN

Supplement: Source data 1. [file elife-73524-data1.zip › crop_w.b/mESC_5.pdf]

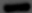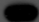

Supplement: Source data 1. [file elife-73524-data1.zip › crop_w.b/mESC_2.pdf]

55 -

40 -

35 -

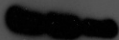

← GAPDH

Supplement: Source data 1. [file elife-73524-data1.zip › crop_w.b/mESC_3.pdf]

1

1

100

11111

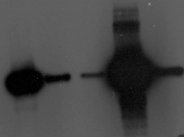

← SUH2

1

Supplement: Source data 1. [file elife-73524-data1.zip › crop_w.b/mESC_1]

1

15

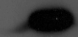

1

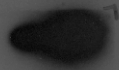

H3

1

1

Supplement: Source data 1. [file elife-73524-data1.zip › crop_w.b/mESC_8.pdf]

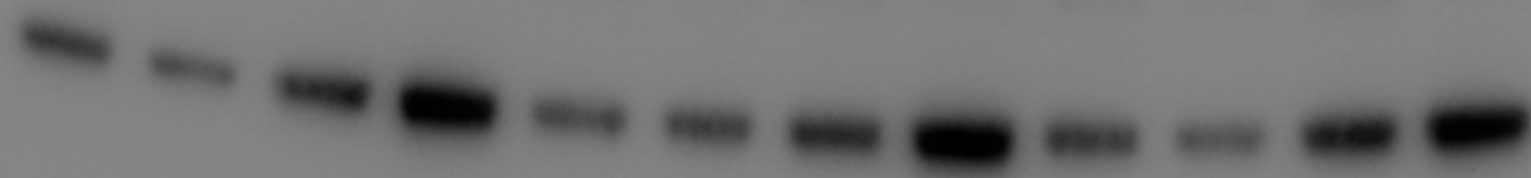

Supplement: Source data 1. [file elife-73524-data1.zip › crop_w.b/brain_12.pdf]

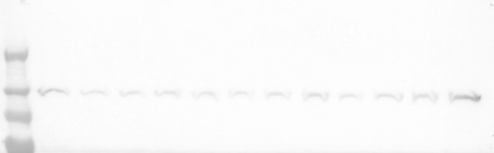

Supplement: Source data 1. [file elife-73524-data1.zip › crop_w.b/kidney_2.pdf]

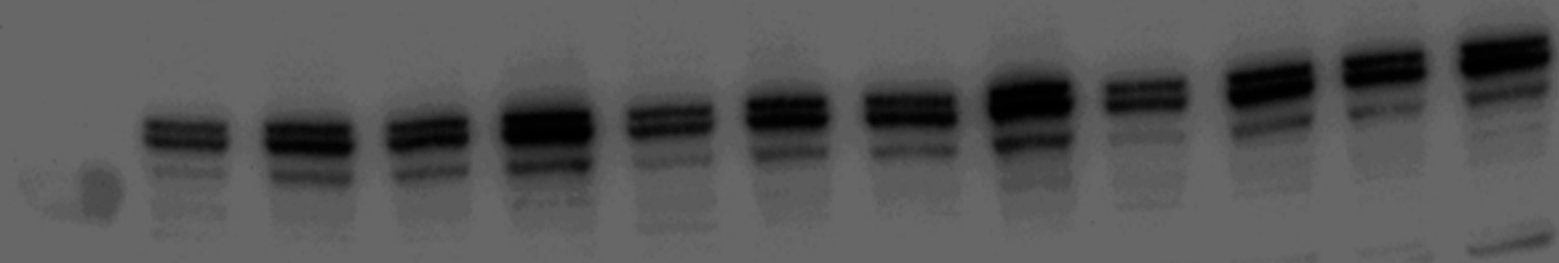

Supplement: Source data 1. [file elife-73524-data1.zip › crop_w.b/brain_13.pdf]

35

43

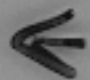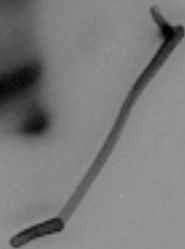

Supplement: Source data 1. [file elife-73524-data1.zip › crop_w.b/brain_11.pdf]

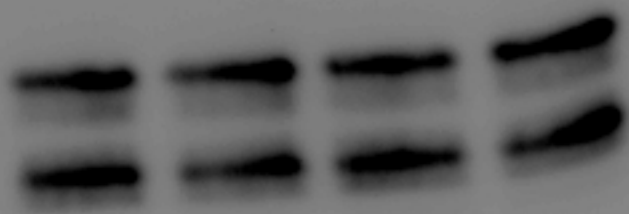

Supplement: Source data 1. [file elife-73524-data1.zip › crop_w.b/liver_8.pdf]

250

-

130

-

100

-

70

-

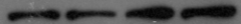

Supplement: Source data 1. [file elife-73524-data1.zip › crop_w.b/kidney_1.pdf]

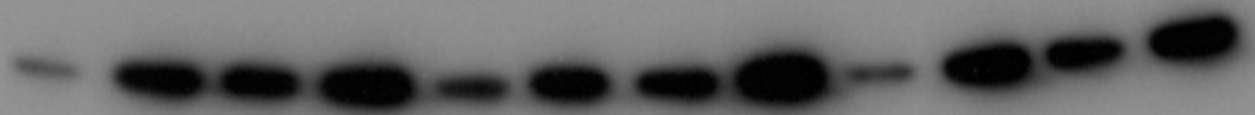

Supplement: Source data 1. [file elife-73524-data1.zip › crop_w.b/brain_10.pdf]

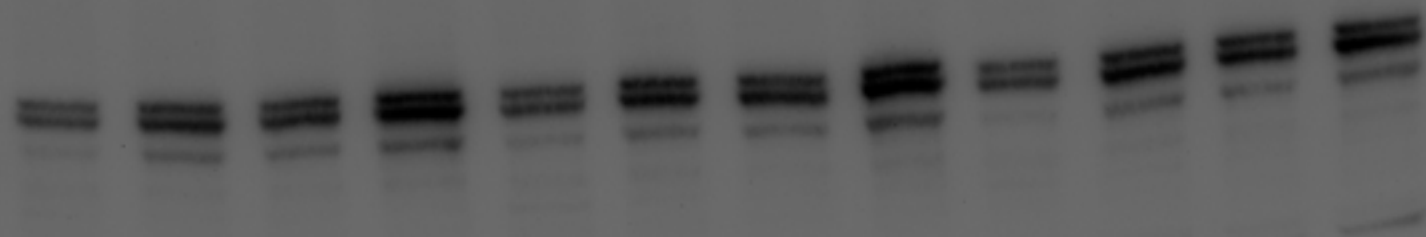

Supplement: Source data 1. [file elife-73524-data1.zip › crop_w.b/brain_14.pdf]

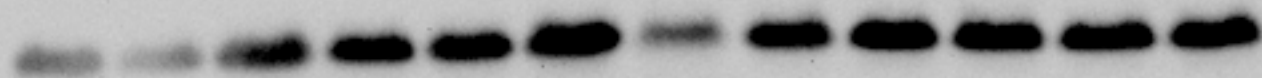

Supplement: Source data 1. [file elife-73524-data1.zip › crop_w.b/heart_8.pdf]

7

7

25°

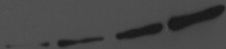

15°

7

7041

Supplement: Source data 1. [file elife-73524-data1.zip › crop_w.b/kidney_4.pdf]

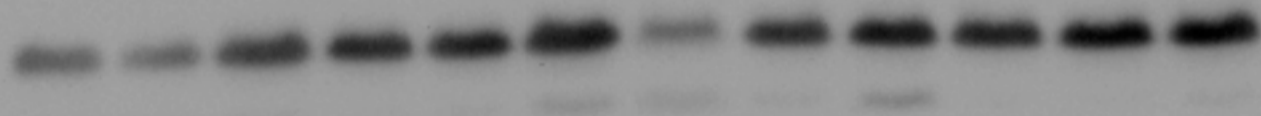

Supplement: Source data 1. [file elife-73524-data1.zip › crop_w.b/heart_9.pdf]

43  
34  
26

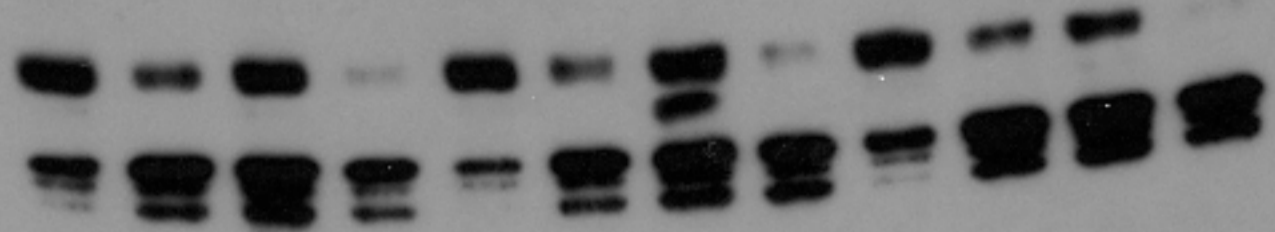

← RNF2 ✓

Supplement: Source data 1. [file elife-73524-data1.zip › crop_w.b/brain_15.pdf]

✓

7

180

130

85

-  
-  
-

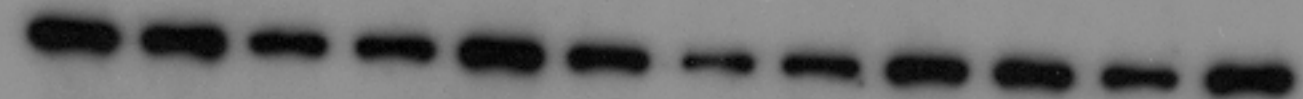

←

ENF20

Supplement: Source data 1. [file elife-73524-data1.zip › crop_w.b/brain_17.pdf]

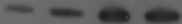

Supplement: Source data 1. [file elife-73524-data1.zip › crop_w.b/brain_8.pdf]

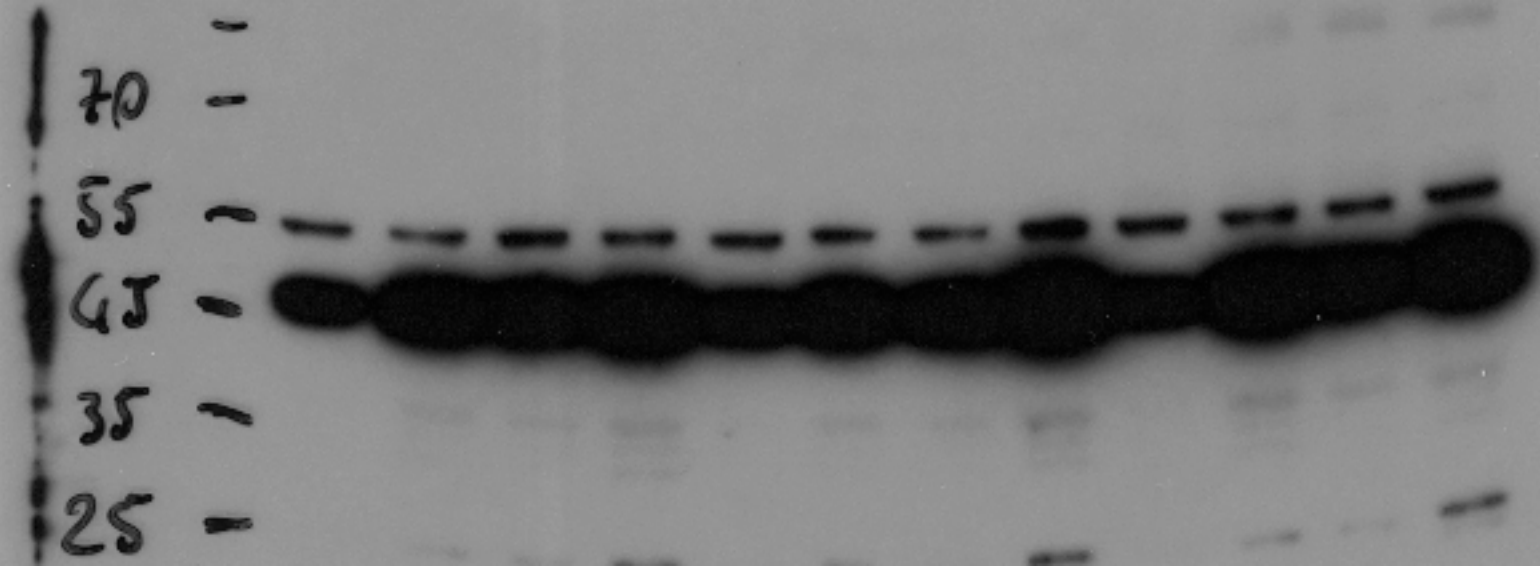

← +12A2.1

≈ 40/42 kDa

Supplement: Source data 1. [file elife-73524-data1.zip › crop_w.b/brain_9.pdf]

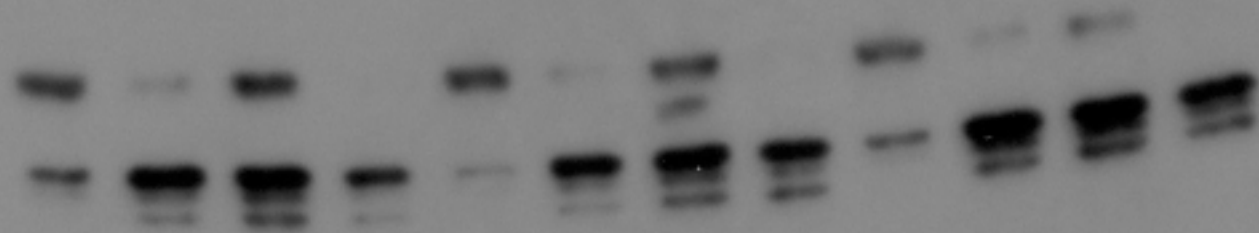

Supplement: Source data 1. [file elife-73524-data1.zip › crop_w.b/brain_16.pdf]
