# Supplementary material for: Distinct and diverse chromatin proteomes of ageing mouse organs reveal protein signatures that correlate with physiological functions
Source: eLife. 2022 Mar 8;11:e73524. doi: 10.7554/eLife.73524 (PMC8933006; doi:10.7554/eLife.73524)

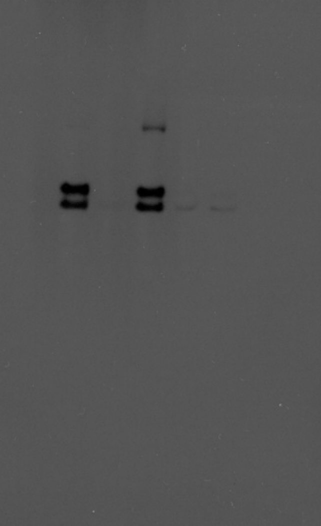

Supplement: Source data 1. [file elife-73524-data1.zip › crop_w.b/mESC_6.pdf]
